# Opportunities for cancer prevention at syringe services programs: acceptability of HPV self-sampling and vaccination among people who inject drugs

**DOI:** 10.1186/s12954-024-00982-3

**Published:** 2024-03-27

**Authors:** Samuel Hinkes, Katrina Ciraldo, Erin Kobetz, Tyler S. Bartholomew, Sarah Rinehart, Nicolette Siringo, Rebecca Barnett, Neha Godbole, Frantzia Jeanty, Morgan Frederick, Hansel E. Tookes

**Affiliations:** 1 Department of Medical Education, University of Miami Miller School of Medicine, Miami, FL USA; 2 Department of Obstetrics & Gynecology, University of Miami Miller School of Medicine, Miami, FL USA; 3 Division of Medical Oncology, Department of Medicine and Public Health Sciences, University of Miami Miller School of Medicine, Miami, FL USA; 4 Department of Public Health Sciences, University of Miami Miller School of Medicine, Miami, FL USA; 5 Division of Infectious Diseases, Department of Medicine, University of Miami Miller School of Medicine, Miami, FL USA

**Keywords:** People who inject drugs, Human papillomavirus, Self-sampling, HPV vaccination, Cancer prevention, Syringe services programs

## Abstract

**Introduction:**

Despite having a high risk of acquiring sexually transmitted infections, people who inject drugs (PWID) often do not receive recommended HPV screenings due to barriers to healthcare. Guideline-based cervical HPV screening and vaccination can prevent cervical cancer. Low-cost, low-barrier methods for cancer screening and prevention are important for vulnerable communities such as PWID.

**Methods:**

We examined acceptability of HPV self-sampling at a syringe services program (SSP). Participants with a cervix (*n* = 49) participated in patient education followed by a survey to assess willingness to perform HPV self-sampling versus standard of care.

**Results:**

59% found self-sampling to be acceptable, citing privacy, ease, and quickness. Among those opting for HPV screening delivered by a provider (*n* = 16), participants cited concerns about adequate sampling (81%) and test accuracy (75%). Notably, only 18% of participants reported complete HPV vaccination.

**Conclusion:**

Cervical HPV self-sampling was acceptable to PWID. SSP-based efforts to provide preventative health services could place tools for cancer screening into the hands of PWID, a need-to-reach community.

**Supplementary Information:**

The online version contains supplementary material available at 10.1186/s12954-024-00982-3.

## Introduction

People who inject drugs (PWID) have limited access to preventive health services in traditional healthcare settings, including recommended cancer screening and HIV, hepatitis C, and sexually transmitted infection (STI) prevention and treatment [1]. Access is worse in states that have not expanded Medicaid, including Florida [2]. PWID are at high risk for contracting STIs, and Miami has the highest incidence of HIV of any U.S. city [3, 4]. Contributors to increased risk for STI acquisition in PWID include concurrent sexual partners, sex work, sexual coercion and assault, and condomless intercourse [4, 5]. 

Syringe services programs (SSP) have played a critical role in sexual health, including decreasing HIV and hepatitis C infections among PWID by as much as 50%.[6] The IDEA SSP in Miami is Florida’s first legal SSP in operation since 2016 [7]. In partnership with the Florida Department of Health, the SSP distributes condoms and screens for and treats HIV, hepatitis C, syphilis, gonorrhea and chlamydia. SSPs are ideal locations to offer such screening to this need-to-reach community because they are frequented by PWID. Despite their proven record of decreasing mortality from cervical cancer, HPV and pap testing are received less often by PWID [8]. Resultantly, PWID have been shown to be more likely to present with more advanced cervical dysplasia and even cancer [8]. 

HPV self-sampling has been shown to be as effective as clinician-collected samples for cervical cancer screening [9]. Self-sampling has been shown in studies to increase rates of cervical cancer screening in high-risk and vulnerable populations and could be offered outside of a traditional clinical setting, including SSPs.^10^ A growing body of evidence has emerged to describe the unmet sexual and reproductive health needs of PWID, suggesting these services would be valuable at an SSP [11]. As such, the primary objective of this study was to assess acceptability of cervical HPV self-swabs. We sought to identify (1) the proportion of PWID who would choose HPV self-sampling versus provider-collected specimens, (2) the reasons for or against choosing HPV self-sampling, (3) sexual practices within our sample and (4) the HPV vaccination rate.

## Methods

The study was conducted at the IDEA SSP in Miami, FL. Participants were recruited at the SSP between April and September 2022. Participants were eligible if they had injected drugs in the last 30 days, had ever been sexually active, and had a cervix.

After pre-screening and verbal informed consent, the survey began with sociodemographic questions. A gender-inclusive sexual behavior survey characterized risk, including years of vaginal sex, number of sexual partners, sex in exchange for money or drugs, sex with another PWID, and lack of complete HPV vaccination series.

All participants born with a cervix identified as cisgender females. We will use the terms ‘born with a cervix’ or ‘cisgender females’ to refer to these participants. An acceptability survey for HPV cervical self-sampling was completed. The survey began with a brief explanation of cervical HPV, clinical presentation, annual incidence of cervical cancer, and associated risk factors. Then, a brief explanation of clinician-provided HPV tests and self-sampling with brief instructions and visual aids were provided. Any questions about this screening method were answered at the time of the survey.

Participants indicated whether they would opt for HPV self-sampling versus standard of care. Participants were then asked to answer the extent to which they agreed with five pre-populated justifications for their choice adapted from a previous acceptability survey [12]. 

All participants received $10 in cash for their participation, which is routinely offered for survey participation at our program. Study data were de-identified, collected, and managed using REDCap electronic data capture tools.^13^ Participants were invited to inquire about study results upon request.

For our descriptive analysis, frequencies and percentages for the sample of those born with a cervix were calculated to describe the participants’ characteristics, sexual behaviors, HPV vaccine history and their responses to acceptability items. Statistical analysis was conducted using SAS software, Version 9.4 (SAS Institute Inc., Cary, NC, USA).

The study protocol was approved by the University of Miami Institutional Review Board (IRB# 20,210,701) and the Sylvester Cancer Center Protocol Review and Monitoring Committee.

## Results

Forty-nine eligible SSP participants completed the survey, and their responses were analyzed. Initially, a broader data collection was conducted including 100 participants identifying as both male and female. For this report and analysis, however, only the 49 cisgender female participants were studied. Three (6.1%) participants were excluded in some analyses due to missing demographic information (Table 1) for a final analytic sample of 46.

**Table 1:** Group characteristics & cervical self-sampling acceptability results (*N* = 46)

**Age Group**	
18 to 29 years old	4 (8.7)
30 to 39 years old	20 (43.5)
40 to 49 years old	16 (34.8)
50 or older	6 (13.0)
**Race/Ethnicity**	
Hispanic	26 (56.5)
Non-Hispanic White	19 (41.3)
Non-Hispanic Black/African American	1 (2.2)
**Educational Attainment (** ***n*** ** = 43)**	
Nursery to 8th grade	2 (4.7)
Some high school	7 (16.3)
High school GED	18 (41.9)
Some College	11 (25.6)
College Graduate	5 (11.6)
**Marital Status (** ***n*** ** = 34)**	
Married / Partnered	7 (20.6)
Widowed, Divorced, Separated	3 (8.8)
Single	24 (70.6)
**Homelessness**	
Housed	17 (37.0)
Unhoused	29 (63.0)
**Has received a full schedule of HPV vaccinations**	8 (18.2)
	**Total, n (%)**
**I would choose the HPV self-sampler because…**	27 (58.7)
**It would allow more privacy than the Pap smear** (***n* = 29)**	
Agree	27 (100)
Neutral	0 (0)
Disagree	0 (0)
**It would be easier to perform than the Pap smear (** ***n*** ** = 29)**	
Agree	24 (88.9)
Neutral	2 (7.4)
Disagree	1 (3.7)
**I have had discomfort with the Pap smear**	
Agree	17 (63.0)
Neutral	4 (14.8)
Disagree	6 (22.2)
**It would be faster than the Pap smear**	
Agree	26 (96.3)
Neutral	1 (3.7)
Disagree	0 (0)
**My provider did not offer cervical cancer screening**	
Agree	1 (3.7)
Neutral	7 (25.9)
Disagree	19 (70.4)
**I would NOT choose the HPV self-sampler because…**	16 (34.8)
**I would not feel confident performing the test myself to get a good sample**	
Agree	13 (81.3)
Neutral	1 (6.3)
Disagree	2 (12.5)
**I’m concerned about the accuracy of the test**	
Agree	12 (75.0)
Neutral	1 (6.3)
Disagree	3 (18.8)
**I’m concerned about feeling pain or discomfort**	
Agree	8 (50.0)
Neutral	2 (12.5)
Disagree	6 (37.5)
**I’m not concerned about my risk of having HPV (e.g., have had HPV vaccine, other reason)**	
Agree	7 (43.8)
Neutral	1 (6.3)
Disagree	8 (50.0)
**My provider offers cervical cancer screening**	
Agree	10 (62.5)
Neutral	2 (12.5)
Disagree	4 (25.0)

Only 18% of participants had received a full schedule of HPV vaccinations (Table 1). Not one of the participants with HIV (*n* = 9) had received full HPV vaccination. The mean number of years since vaginal coitarche was 24.4. About half of the participants (51%) reported having sex with partners of more than one gender identity. Many participants endorsed condomless sex (58%).

Of the 46 participants, 59% would choose cervical HPV self-sampling over screening delivered by a provider (Table 1). Respondents tended to opt for cervical self-sampling due to privacy, ease, and quickness (100%, 89%, and 96%, respectively). 63% reported a history of discomfort with the pap smear. Respondents who would choose provider-delivered screening (*n* = 16) cited concern about getting an adequate sample themselves (81%) and concern about accuracy of the test (75%). Others were concerned about pain or discomfort with self-sampling (50%).

## Discussion

We found acceptability of cervical HPV self-sampling among PWID, though not as substantially as prior studies of non-PWID and other hard-to-reach populations [12, 14, 15]. These findings may be explained by an element of mistrust with the healthcare system at large and suggest that with built rapport, SSPs may be able to offer these services to PWID and reduce disparities in receipt of preventative health services.

The Centers for Disease Control (CDC) and Advisory Committee on Immunization Practices (ACIP) did not recommend HPV vaccination for adults aged 27–45 across the board. Instead, they endorse shared decision making between patient and provider. The American College of Obstetrics & Gynecology released a statement in June 2019 supporting the position of the ACIP [16]. Most of our SSP participants fall within this age range and could benefit from this approach to cancer prevention. Implementation of an HPV prevention program at SSPs would likely require partnership with healthcare providers to coordinate close follow-up for patients positive for high-risk HPV. Our study allowed us to survey PWID at an SSP, an institution which many have come to trust to deliver culturally competent STI screening (e.g., HIV, gonorrhea, chlamydia, etc.) and a place where they may share their substance use and sexual behaviors more openly. This setting presents a potentially valuable venue to offer preventive and reproductive health services (e.g., Pap smears, STI screening, PrEP services, etc.) for people with a high risk of STI acquisition in a one-stop shop [17]. 

These findings demonstrate that this low-cost, low-barrier method of cancer screening is acceptable among PWID who avoid the traditional healthcare system due to intransigent stigma [18, 19]. PWID are willing to receive harm reduction services and preventive healthcare at SSPs, which have historically served this vulnerable and stigmatized population. As PWID are at increased risk for high-risk—often concomitant—HPV strains and subsequent dysplasia [20], this drives the need to provide screening services to this unique community. Our findings suggest that SSP participants might utilize HPV screening programs if they could be incorporated into low barrier SSP settings.

Screening services would especially protect PWID living with HIV as HPV is more likely to evade immune responses with HIV co-infection. Some scholars assert that self-sampling outside the supervision of a provider without a proper physical examination leaves room to miss cervical dysplasia. Others contend that individual interventions to increase health literacy and patient engagement may foster understanding of self-sampling as a tool to detect a risk factor for cancer and preventable illness but not cancer itself [21]. 

This study had limitations. While this study demonstrated acceptability, this study also does not assess linkage to care or ways to pay for self-sampling. Also, the acceptability survey was limited to five multiple-choice options for each selection which may fail to capture the breadth of responses and influences for this population, including health literacy and familiarity with HPV screening. Finally, while we wished to characterize the acceptability of self-sampling in the setting of an SSP, we did not specifically ask whether they would be willing to do the self-sampling at the SSP.

Future efforts should continue to characterize the risk of HPV for PWID to craft more appropriate screening recommendations. Additionally, studies should include a community-based participatory research approach, including PWID in the design and implementation of preventive health services at SSPs according to their priorities. Prior research has demonstrated the feasibility of providing low-barrier reproductive healthcare to women who inject drugs at SSPs but did not characterize the uptake or acceptability of HPV vaccination among PWID [22]. The prospect of self-sampling might allow for more privacy and ease for the patient and would also present an opportunity for HPV vaccination up to age 45 as an additional means of prevention. PWID have demonstrated resilience in acquisition of valuable preventive care and harm reduction in the setting of SSPs, care that could include sexual and reproductive health services, including HPV self-sampling and vaccination.

### Electronic supplementary material

Below is the link to the electronic supplementary material.

Supplementary Material 1

## Data Availability

The datasets used and/or analyzed during the current study are available from the corresponding author on reasonable request [see Additional File 1].

## Abbreviations

PWID: people who inject drugs
STI: sexually transmitted infection
SSP: syringe services program
HPV: human papillomavirus
IDEA: Infectious Disease Elimination Act
HIV: human immunodeficiency virus
