# Supplementary material for: Opportunities for cancer prevention at syringe services programs: acceptability of HPV self-sampling and vaccination among people who inject drugs
Source: Harm Reduct J. 2024 Mar 27;21:70. doi: 10.1186/s12954-024-00982-3 (PMC10967053; doi:10.1186/s12954-024-00982-3)
Supplement: Supplementary file 1 — Supplementary Material 1 [file 12954_2024_982_MOESM1_ESM.docx]

**Additional Files**

**Additional File 1**

.csv file

REDCap Exported Data Report

This file contains all de-identified data points for all research collected for this study. Please note some of the data responses are for anal self-sampling, as this was also collected for future research purposes.

**Additional File 2**

.docx file

Survey Designer and Codebook

This file contains the original survey structure, including variable names. This should serve as a guide to understand the REDCap Exported Data Report if necessary. Please note some of the data responses are for anal self-sampling, as this was also collected for future research purposes.
